# Identification of a measles variant displaying mutations impacting molecular diagnostics, Geneva, Switzerland, 2023

**DOI:** 10.2807/1560-7917.ES.2024.29.5.2400034

**Published:** 2024-02-01

**Authors:** Francisco-Javier Pérez-Rodríguez, Pascal Cherpillod, Valentine Thomasson, Pauline Vetter, Manuel Schibler

**Affiliations:** 1 Laboratory of virology, Laboratory Medicine Division, Geneva University Hospitals, Geneva, Switzerland; 2 National Measles and Rubella Reference Laboratory (CNRRR), Geneva University Hospitals, Geneva, Switzerland; 3 Geneva Center for Emerging Viral Diseases, Geneva University Hospitals, Geneva, Switzerland

**Keywords:** measles, genetic variability, PCR primer mismatch, PCR sensitivity

## Abstract

Real-time PCR is one of the most widely used techniques to diagnose measles cases. Here we report measles virus variants with three genetic mutations in the reverse primer annealing site of a widely used PCR. The mutations result in a slight loss of the PCR sensitivity. Variants bearing the three mutations presently circulate in different countries since at least the end of 2021. Our findings highlight the usefulness of molecular surveillance in monitoring if oligonucleotides in diagnostic tests remain adequate.

Measles diagnostic relies on viral RNA detection by real-time reverse-transcription-PCR (RT-rPCR), in combination with virus-specific IgM detection. Genetic variability can reduce the sensitivity of a RT-rPCR assay due to primers- and/or probe–template mismatches. Here we describe measles virus variants detected through the Swiss molecular surveillance, which have genetic mutations in the primer annealing site of a commonly employed RT-rPCR.

## Comparing diagnostic-PCR oligonucleotide sequences against viral sequences obtained through genotyping

At the Laboratory of Virology of the Geneva University Hospitals we currently use the United States Centers for Disease Control and Prevention (CDC, Atlanta) RT-rPCR assay, which targets the measles virus nucleoprotein (N) gene [1,2], (further here ‘CDC PCR’). Kits containing the primer and probe oligonucleotides for this assay are provided by the CDC to laboratories belonging to the Global Measles and Rubella Laboratory Network (GMRLN) [1]. The 75-nt region targeted by the assay is part of the 450 nt region in the C-terminal of the N gene (N-450), which is recommended for routine genotyping by the World Health Organization (WHO) [3]. In addition to characterising measles viruses, the genotyping allows to check for any evolution of the assay target in circulating virus variants, by aligning the primer and probe sequences against N-450 sequences of such viruses, that have been deposited in databases such as GenBank [4] or the WHO Global Measles Nt Sequence Database (MeaNS2, https://who-gmrln.org/means2) [5].

## Measles virus sequencing results in Switzerland from January to December 2023

Swiss laboratories are requested to submit samples that are positive for measles RT-rPCR to the National Measles and Rubella Reference Laboratory (CNRRR, Geneva University Hospitals) for sequencing and genotyping [6]. Swiss public health authorities use the data generated by the CNRRR and those provided by physicians to establish epidemiological links between cases and to identify their source of infection [6].

During 2023, we genotyped 36 positive samples by sequencing the N-450 region based on the CDC protocol [1]. Except for one sample collected in December 2022 and corresponding to genotype B3, all sequences belonged to genotype D8. In the MeaNS2 database each unique N-450 sequence is assigned to a distinct sequence identifier (DSId) [7]. The 35 D8 genotype sequences corresponded to eight different DSIds. A sequence alignment of the eight genotype D8 DSIds and of the genotype B3 DSId detected by CNRRR in 2023, together with the primers and probes used in the CDC PCR is shown in the Figure. Alignments presented in this report were performed using Geneious Prime 2021.2.2 (https://www.geneious.com). Representative sequences of each DSId that we found were submitted to GenBank (accession numbers: PP229487–PP229495).

**Figure:**
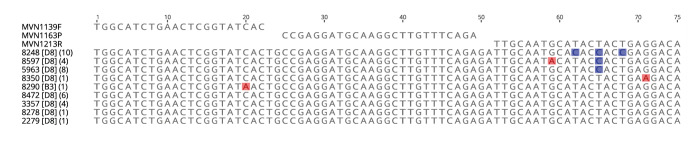
Alignment between CDC measles RT-rPCR primers and probe^a^ and the distinct sequence identifiers (DSIds) detected in Switzerland, January–December 2023 (n = 9 DSIds)

CDC: United States Centers for Disease Control and Prevention; DSId: distinct sequence identifiers; RT-rPCR: real-time reverse-transcription-PCR.

MVN1139F: forward primer; MVN1163P: probe; MVN1213R: reverse primer (shown as reverse complement).

^a^ The assay is described in [1].

The genotype is indicated in square brackets and the number of cases associated with each DSId in parentheses. The alignment was performed using Geneious Prime 2021.2.2.

## Detection of mismatches in a primer annealing site, and impact on the sensitivity of the measles virus detection assay

The alignment between the different N-450 sequences obtained in 2023 by the CNRRR and the sequences of the oligonucleotides used in the CDC PCR revealed three mismatches between one DSIds (8248) belonging to genotype D8 and the reverse primer (MVN1213R) (Figure). A total of 10 cases were affected by measles virus strains characterised by this DSIds. In four of these cases a recent travel was reported (to Russia, Kazakhstan, Saudi Arabia and Italy). The three mutations are T-to-C synonymous transitions.

To investigate the impact of these three mismatches on the sensitivity of the test, we performed serial dilutions of two positive samples: one containing the three mismatches (sample A, DSId 8248) and one without mismatches (sample B, DSId 8278). RNA extraction from each dilution was performed in duplicate and each eluate was tested in duplicate. Both samples were tested in parallel with the CDC PCR and with the assay published in 2005 by El Mubarak et al. (comparator PCR) [8], according to the conditions validated in our laboratory. For the CDC PCR the primers and probe were used with the AgPath-ID One-Step RT-PCR Reagents (ThermoFisher Scientific) and the following RT-PCR programme: 50 °C for 15 min, 95 °C for 10 min and 40 cycles (95 °C for 8 s, 60 °C for 34 s). The comparator PCR primers and probe were used with QuantiTect Probe RT-PCR Kit (Qiagen) and the following programme: 50 °C for 30 min, 95 °C for 15 min and 45 cycles (94 °C for 15 s, 60 °C for 1 min). The comparator PCR target sequences, obtained during sequencing of the N-450 region, were identical for samples A and B.

For sample A, the maximum dilution for which all replicates were positive was 10 ^− 2^ for the CDC PCR and 10 ^− 3^ for the comparator PCR (Table 1). For sample B, the maximum dilution positive for the four replicates was 10 ^− 4^ for the CDC PCR and 10 ^− 3^ for the comparator PCR (Table 1). These results suggest a partial loss of sensitivity of the CDC PCR in detecting the sample with the three mutations.

**Table 1:** Results of the CDC and comparator PCRs carried out in parallel on 10-fold serial dilutions of two samples with respectively (A) mismatches to the CDC PCR reverse primer^a^ and (B) no mismatches to this primer^b^, Switzerland, 2023 (n = 2 samples)^c^

Dilution	Sample A DSId 8248 (three mismatches)				Sample B DSId 8278 (no mismatches)			
	CDC PCR		Comparator PCR		CDC PCR		Comparator PCR	
10 ^− 1^	+ / +	+ / +	+ / +	+ / +	n.t.	n.t.	n.t.	n.t.
10 ^− 2^	+ / +	+ / +	+ / +	+ / +	+ / +	+ / +	+ / +	+ / +
10 ^− 3^	**-**/**-**	**-**/ +	+ / +	+ / +	+ / +	+ / +	+ / +	+ / +
10 ^− 4^	+ /**-**	**-**/**-**	**-**/**-**	**-**/**-**	+ / +	+ / +	+ / +	**-**/**-**
10 ^− 5^	**-**/**-**	**-**/**-**	**-**/**-**	**-**/**-**	**-**/**-**	+ /**-**	**-**/**-**	**-**/**-**
10 ^− 6^	n.t.	n.t.	n.t.	n.t.	**-**/**-**	**-**/**-**	**-**/**-**	**-**/**-**

CDC: United States Centers for Disease Control and Prevention; DSId: distinct sequence identifier; n.t.: not tested; +: positive PCR result; -: negative PCR result.

^a^ Sample A has a sequence (DSId 8248) presenting three mismatches to the reverse primer of the CDC PCR in the annealing site of this primer.

^b^ Sample B has a sequence (DSId 8278) with no mismatches to the reverse primer of the CDC PCR in the annealing site of this primer.

^c^ Samples A and B share the same sequence in the region targeted by the comparator PCR.

RNA extraction from each dilution was performed in duplicate and each eluate was tested in duplicate. Each cell of the table corresponds to one extraction duplicate. Maximum dilutions positive for the four replicates are highlighted.

## Temporal and spatial distribution of sequences containing the three mutations

A search for N-450 sequences belonging to the D8 genotype was performed in MeaNS2 database on 03 January 2024, using case onset dates from 01 January 2021 to 13 December 2023. We obtained a total of 2,682 results classifiable into 240 DSIds. The three described mutations were found in 451 sequences (16.8%) belonging to 28 DSIds. Cases showing such mutations appeared for the first time in December 2021 and were from Tajikistan. Since then, sequences containing these mutations have been deposited in the database from 18 northern hemisphere countries from European, Eastern Mediterranean, Americas, and Western Pacific WHO regions (Table 2, only partial data are shown). Although the number of sequences containing such mutations appears to be decreasing from May 2023, cases continue to be detected (Table 2). A GenBank search revealed no additional sequences.

**Table 2:** Monthly distribution by country of genotype D8 sequences containing the three described mutations in MeaNS2, January 2021–December 2023 (n = 373 sequences)

Year-Month	Country^a^ Number of sequences								
	TJ^a^	RU^a^	BY^a^	SE^a^	CH^a^	US^a^	UK^a^	FI^a^	DE^a^
2021–12	5	0	0	0	0	0	0	0	0
2022–01	0	3	0	0	0	0	0	0	0
2022–02	1	0	0	0	0	0	0	0	0
2022–03	0	2	0	0	0	0	0	0	0
2022–04	0	2	0	0	0	0	0	0	0
2022–05	0	0	0	0	0	0	0	0	0
2022–06	0	3	0	0	0	0	0	0	0
2022–07	0	0	0	0	0	0	0	0	0
2022–08	0	2	0	0	0	0	0	0	0
2022–09	0	0	0	0	0	0	0	0	0
2022–10	0	4	0	0	0	0	0	0	0
2022–11	5	2	0	0	0	0	0	0	0
2022–12	2	23	0	0	0	0	0	0	0
2023–01	0	30	0	0	0	0	0	0	0
2023–02	0	37	1	0	0	0	0	0	0
2023–03	0	62	1	1	0	0	0	0	0
2023–04	0	44	5	0	1	1	0	0	0
2023–05	0	50	2	0	5	0	3	0	0
2023–06	0	17	7	0	1	0	2	0	0
2023–07	0	11	4	2	0	0	1	1	1
2023–08	0	0	1	0	0	0	0	0	0
2023–09	0	0	0	0	0	1	3	0	0
2023–10	0	0	0	0	0	0	7	0	1
2023–11	0	0	0	0	3	0	13	0	0
Total	13	292	21	3	10	2	29	1	2

MeaNS2: World Heath Organization Global Measles Nt Sequence Database.

^a^ BY: Belarus; CH: Switzerland; DE: Germany; FI: Finland; RU: Russia; SE: Sweden; TJ: Tajikistan; UK: United Kingdom; US: United States.

Only results from countries that have authorised the use of their data are shown.

The search was limited to N-450 sequences. When case onset date was unavailable, the date of collection of the sample was used. Otherwise, the date of reception of the sample was used.

## Discussion

Through genotyping measles viruses by sequencing the N-450 region of their genomes, we discovered three mutations in the annealing site of the reverse primer used in the CDC RT-rPCR [1,2]. This PCR is widely used to detect the presence of measles viral genome in clinical specimens, thereby supporting the diagnosis of patients and molecular surveillance. A rapid assessment of the identified mutations’ impact on the RT-rPCR suggests a partial loss of sensitivity, which could result in false negative results for samples with low viral load.

A search of the MeaNS2 database revealed that the three mutations are present in viruses corresponding to more than 25 different DSIds that have been reported since December 2021 in 18 countries. Although it appears as if numbers of viruses bearing these mutations in the database are declining, their circulation continues. Even though we did not observe a great loss of sensitivity, it cannot be ruled out that additional mutations in the N gene could lead to variants not detectable by the CDC assay.

To minimise the chances of false negatives, the primer sequences could be updated to consider the observed mutations (e.g. by designing a degenerate reverse primer). Alternatively, another set of oligonucleotides annealing to different targets could be added to the assay, either in the same or in different wells. This strategy allows a positive result to be obtained even if a target fails to amplify [9].

In terms of verifying the continued appropriateness of the molecular diagnostic tests to detect current measles strains, it is advantageous if the sequences of the primers and probes are known, and if the tests target one of the most frequently sequenced regions of the measles virus genome for genotyping or surveillance purposes (N-450, the measles fusion (MF)-non-coding region and the haemagglutinin gene). For tests targeting other regions of the genome, the increasing implementation of whole genome sequencing of viruses may be beneficial to detect mutations potentially affecting assay performance. Unfortunately, whole genome sequencing techniques are challenging and not available in many reference laboratories yet. It is also noteworthy that to assess the primers’ and probe’s continued match to circulating strains, employing in-house PCRs is of value, as this allows knowledge of the sequences of oligonucleotides used. Such information may not be disclosed in commercial tests, hampering its inclusion for the understanding and monitoring of sensitivity.

A limitation of the current study is that the number of samples tested for the sensitivity was low. However, this should not bear on the main messages of this report. In addition, associated epidemiological data have not been examined, beyond the travel information of the cases detected in Switzerland.

### Conclusion

Measles molecular surveillance is useful to track its transmission routes, characterise outbreaks and distinguish between imported cases and cases due to endemic circulation of the virus. The results of the current work reinforce the additional relevance of this type of surveillance for monitoring whether oligonucleotides used in molecular diagnostic tests remain adequate/up to date. Our findings also raise awareness of measles virus variants with three genetic mutations in the annealing site of the CDC PCR reverse primer, resulting in a slight loss of this test’s sensitivity. The variants presently circulate in different countries at least since the end of 2021.
